# Association of laboratory test results with the bleeding history in patients with inherited platelet function disorders (the Bleeding Assesment Tool - LABoratory tests substudy): communication from the Platelet Physiology ISTH-SSC

**DOI:** 10.1016/j.rpth.2023.102305

**Published:** 2023-12-20

**Authors:** Paolo Gresele, Emanuela Falcinelli, Loredana Bury, Marie-Christine Alessi, Giuseppe Guglielmini, Céline Falaise, Gianmarco Podda, Mathieu Fiore, Francesco Mazziotta, Teresa Sevivas, Nuria Bermejo, Erica De Candia, Meera Chitlur, Michele P. Lambert, Luca Barcella, Ana C. Glembotsky, Marie Lordkipanidzé

**Affiliations:** 1 Section of Internal and Cardiovascular Medicine, Department of Medicine and Surgery, University of Perugia, Perugia, Italy; 2 Centre for CardioVascular and Nutrition Research, Institut National de la Sante et de la Recherche Medicale 1263, Institut National de la Recherche Agronomique 1260, Marseille, France; 3 Dipartimento di Scienze della Salute, Medicina III, Azienda Socio Sanitaria Territoriale Santi Paolo e Carlo, Università degli Studi di Milano, Milano, Italy; 4 Laboratory of Hematology, University Hospital of Bordeaux, Pessac, France; 5 Serviço de Sangue e Medicina Transfusional, Centro Hospitalar e Universitario de Coimbra, Coimbra, Portugal; 6 Hospital San Pedro de Alcantara, Caceres, Spain; 7 Hemostasis and Thrombosis Unit, Policlinico Universitario Agostino Gemelli Istituto di Ricovero e Cura a Carattere Scientifico, Roma, Italy; 8 Department of Translational Medicine and Surgery, Università Cattolica del Sacro Cuore, Roma, Italy; 9 Children’s Hospital of Michigan, Detroit, Michigan, USA; 10 Children’s Hospital of Philadelphia, Philadelphia, USA; 11 Immunohematology and Transfusion Medicine & Hemostasis and Thrombosis Center, Azienda Socio Sanitaria Territoriale Papa Giovanni XXIII, Bergamo, Italy; 12 Instituto de Investigaciones Médicas A. Lanari, Facultad de Medicina, Universidad de Buenos Aires, Buenos Aires, Argentina; 13 Departamento Hematología Investigación, Consejo Nacional de Investigaciones Científicas y Tecnológicas, Universidad de Buenos Aires, Instituto de Investigaciones Médicas, Buenos Aires, Argentina; 14 Université de Montréal, Montréal, Québec, Canada; 15 Research Center, Montreal Heart Institute, Montréal, Québec, Canada

**Keywords:** blood platelets, hemorrhage, inherited platelet function disorders, ISTH-BAT bleeding score, platelet function tests

## Abstract

**Background:**

In hemophilia and von Willebrand disease, the degree of alteration of laboratory assays correlates with bleeding manifestations. Few studies have assessed the predictive value for bleeding of laboratory assays in patients with inherited platelet function disorders (IPFDs).

**Objectives:**

To assess whether there is an association between platelet function assay results and bleeding history, as evaluated by the International Society on Thrombosis and Haemostasis (ISTH) bleeding assessment tool (BAT).

**Methods:**

Centers participating in the international ISTH-BAT validation study were asked to provide results of the diagnostic assays employed for the patients they enrolled, and the association with the individual patients’ bleeding score (BS) was assessed.

**Results:**

Sixty-eight patients with 14 different IPFDs were included. Maximal amplitude of platelet aggregation was significantly lower in patients with a pathologic BS and correlated inversely with the BS, a finding largely driven by the subgroup of patients with Glanzmann thrombasthenia and CalDAG-GEFI deficiency; after their exclusion, TRAP-induced aggregation remained significantly lower in patients with a pathologic BS. Bleeding time was significantly more prolonged in patients with a high BS than in those with a normal BS (27.1 ± 6.2 minutes vs 15.1 ± 10.6 minutes; *P* < .01). Reduced α-granule content was significantly more common among patients with a pathologic BS than among those with a normal BS (80% vs 20%; *P* < .05). Receiver operating characteristic curve analysis revealed a significant discriminative ability of all the aforementioned tests for pathologic BS (*P* < .001), also after exclusion of patients with Glanzmann thrombasthenia and CalDAG-GEFI deficiency.

**Conclusion:**

This study shows that altered platelet laboratory assay results are associated with an abnormal ISTH-BAT BS in IPFD.

## Essentials

• The association of bleeding severity with platelet laboratory test results is poorly studied.
• We assessed this association in patients with inherited platelet function defects.
• Alterations of some platelet laboratory assays were associated with bleeding severity.
• Integration of bleeding assessment tools with laboratory assays may help in patient management.

## 1 Introduction

Inherited platelet function disorders (IPFDs) are hemorrhagic diseases characterized by defective platelet activation and sometimes reduced platelet number, associated with mild to severe mucocutaneous bleeding. Although IPFDs make up a significant fraction of all congenital bleeding disorders [1,2], they remain poorly characterized and difficult to diagnose [3].

A diagnostic approach based on careful clinical evaluation and a streamlined panel of appropriately selected laboratory assays has been proposed; bleeding history represents an essential step in the diagnostic workup, and its alteration is crucial for the decision to embark on complex and expensive laboratory studies [[4], [5], [6]].

The International Society on Thrombosis and Haemostasis (ISTH) bleeding assessment tool (BAT), initially employed only for patients with suspected von Willebrand disease (VWD), has been recently validated for the assessment of the bleeding history of patients with inherited platelet disorders (IPDs) [7]. Moreover, it has been shown to be highly predictive of postsurgical bleeding in a retrospective study, with patients with IPFD having an ISTH-BAT of ≥6 suffering a significantly higher incidence of postsurgical bleeding [8] and spontaneous bleeding in a prospective study in a cohort of patients with IPFD followed-up for 2 years, with patients showing a high baseline bleeding score (BS) having a significantly greater risk of subsequent hemorrhagic events [9].

The ISTH-BAT has previously been shown to be a predictor of clinical outcome and replacement therapy in adults with VWD, and interestingly, in that study, the incidence of bleeding was inversely related to baseline von Willebrand factor ristocetin cofactor and factor (F)VIII levels: patients with type 1 VWD and low von Willebrand factor ristocetin cofactor (<10 U/dL) and FVIII (<20 IU/dL) were more likely to suffer from bleeding and require replacement therapy during follow-up [10].

In classical hemophilia too, there is a good relationship between plasma levels of FVIII and the frequency and severity of bleeding symptoms [11], and the endogenous thrombin generation potential provides additional insight into the bleeding phenotype [12,13]. On the contrary, it is generally held that in most mild/moderate bleeding disorders, laboratory abnormalities may prove inconsistent in predicting the risk of bleeding [6].

In contrast to VWD or hemophilia, only a few studies have assessed the predictive value of laboratory assays for bleeding manifestations in patients with IPFD, with conflicting results [1,[14], [15], [16], [17], [18], [19]]. One study showed no relationship between the severity of bleeding and platelet dense-granule content or impaired light transmission aggregometry (LTA) in 65 patients with IPFD [1]. Similarly, defective platelet function assessed by lumiaggregometry in platelet-rich plasma was not associated with an enhanced ISTH-BAT BS in 427 subjects with suspected IPFD [14]. Moreover, the degree of reduction of platelet glycoprotein (GP) expression by flow cytometry, as well as decreased lumiaggregometry and α-granule content, were not associated with bleeding severity in 32 patients with a primary secretion defect (PSD) [15].

On the contrary, 1 study in a rather large cohort of individuals with Glanzmann thrombasthenia (GT), Bernard–Soulier syndrome (BSS), and defective aggregation in response to adrenaline reported that a higher ISTH-BAT score was predictive of the presence of a platelet defect on LTA and that, in binary logistic regression, the platelet defect had a statistically significant effect on the ISTH-BAT score model (odds ratio [ORs], 3.25; 95% CI, 2.13-4.37; *P* < .001) [16]. Recently, a single institution study in a small cohort of patients with suspected IPD concluded that abnormal platelet transmission electron microscopy (TEM) is associated with high likelihood of an abnormal ISTH-BAT [18]. Finally, a study involving 37 patients with uncharacterized IPFD showed that reduced maximal platelet aggregation induced by collagen was associated with more bruises and wound healing problems and more severe dense granule deficiency with surgical bleeding [19].

The aim of the current study was to assess if there is an association between the results of the laboratory assays employed for diagnosis and the bleeding manifestations assessed by the ISTH-BAT BS in a series of well-characterized patients with IPFD.

## 2 Methods

### 2.1 Design of the study

This was an ad hoc designed subgroup analysis of the Bleeding Assesment Tool-Validation (BAT-VAL) study, a multicentric international prospective study promoted by the Platelet Physiology Scientific and Standardization Committee of the ISTH including adult and pediatric patients with a diagnosis of IPD confirmed according to well-defined laboratory and/or molecular genetic criteria [7,9] (Supplementary Table S1). Genetic confirmation was available for 56% of patients, but it is worth noting that 70% of patients without a genetic confirmation had a diagnosis of PSD or combined α/δ storage pool disorder, conditions for which a causative gene has not been identified yet. We did not collect detailed information on the reasons for initial referral of the patients to the participating centers.

The results of the laboratory platelet function assays performed for IPFD diagnosis according to the ISTH Scientific and Standardization Committee guidance [20] were retrospectively retrieved from the files of the patients who had been enrolled in the BAT-VAL study [7]. We defined IPFDs as disorders in which platelet dysfunction was the dominant phenotypic feature independent of platelet count [7,9].

All participating investigators were asked to fill a standardized report form by retrieving the results of the platelet function studies carried out for the diagnosis of IPFD (see Supplementary materials "Case report form"). Laboratory data included platelet count, mean platelet volume (MPV), bleeding time (BT), PFA-100 System (Siemens Healthcare Diagnostic) closure time (CT), LTA, α- and dense-granule content and release, platelet GP surface expression by flow cytometry, clot retraction, and serum thromboxane B_2_ (TxB_2_).

Informations concerning method, type of instrument, manufacturer, and concentration of agonist used for platelet activation and internally established normal reference intervals for each test were also collected (Supplementary materials).

Platelet function assay results were classified as abnormal when outside the internal laboratory reference interval. The ISTH-BAT BS was considered abnormal when it was ≥4 for males and ≥6 for females [6,21]. For pediatric patients, the ISTH-BAT BS was considered abnormal when it was ≥3 [21].

### 2.2 Laboratory assays

Details of the laboratory assays and respective normal ranges can be found in Supplementary Table S2. Platelet count and MPV were assessed by different automated hematological counters. Platelet function analyzer (PFA-100) test was performed with the collagen/epinephrine (EPI) and collagen/ADP cartridges, and the CT was recorded [22]. The BT test was performed according to the method of Mielke [23] or Ivy et al. [24]. Platelet aggregation was assessed in most centers by LTA [25] and in one by impedance aggregometry [26]. The concentration and types of agonists used for platelet aggregation testing differed among centers (Supplementary Table S2). Some centers tested more than 1 agonist concentration. In particular, 5 patients were tested with more than 1 dose of EPI, 14 of ADP, 3 of collagen, and 6 of TRAP-6. To correlate LTA results with the BS, we considered the highest agonist concentration tested.

Platelet surface GP expression was measured by flow cytometry with specific fluorescent-conjugated antibodies against GPIIb (CD41), GPIIIa (CD61), GPIbα (CD42b), and GPIX (CD42a). ADP-induced activation of the GPIIb/IIIa complex was assessed by measuring PAC-1 antibody binding by flow cytometry [27]. Platelet α-granule content was assessed by enzyme-linked immunosorbent assay (ELISA) (β-thromboglobulin, plasminogen activator inhibitor-1, fibrinogen of platelet lysates) or immunofluorescence (thrombospondin), while agonist-induced α-granule release was assessed by flow cytometry using an anti-CD62P antibody to test platelet surface P-selectin expression or by ELISA (β-thromboglobulin) in stimulated platelet supernatants after centrifugation [28] (Supplementary Table S2).

Dense-granule content was assessed by lumiaggregometry (adenosine triphosphate), high performance liquid chromatography (serotonin), or fluorescence spectrophotometry (serotonin), while release was assessed by high performance liquid chromatography (serotonin), lumiaggregometry (adenosine triphosphate), or flow cytometry (mepacrine and CD63) [28]. The concentration and types of agonists used for platelet granule secretion testing differed among centers (Supplementary Table S2). Finally, TxB_2_ concentration in serum was measured by ELISA (Supplementary Table S2).

### 2.3 ISTH-BAT BS

The BS was calculated either manually, using the interpretation grid, or automatically, using the web-based version (https://bh.rockefeller.edu/ISTH-BATR), at enrollment in the BAT-VAL study [7].

To evaluate associations between single items of the ISTH-BAT and platelet function laboratory assays, we defined items with a score of >2 as “severe.”

### 2.4 Statistical analysis

Data are reported as medians and 25th to 75th percentiles (IQR) when continuous and as counts and percentages when categorical. Correlations between laboratory variables and the ISTH-BAT BS were assessed with the Pearson correlation coefficient (ρ). Receiver operating characteristic (ROC) curves for diagnostic prediction (abnormal BS) were calculated for each test, and areas under the curve (AUCs), with binomial exact CIs for AUC, sensitivity, specificity, negative predictive value, and positive predictive value, were assessed. Cutoff values for the most relevant comparisons were also calculated using the Youden index.

Test results were used to estimate the likelihood of abnormal BS as ORs with 95% CIs. Relationships between bleeding symptoms and laboratory test results were evaluated using the Mann–Whitney U-test and chi-square test.

Given that 28% of the enrolled patients had GT and CalDAG-GEFI deficiency, conditions typically associated with a high BS and severe platelet dysfunction, which could introduce bias to the overall analysis, a separate analysis was performed excluding these groups.

A 2-sided *P* value of <.05 was considered statistically significant. The IBM SPSS Statistics v.25 software was used for all analyses.

## 3 Results

### 3.1 Patient characteristics

Eleven centers worldwide responded to the survey, and 68 patients were included in this study (34.7% of the initially enrolled IPFD population) [7], 37 of whom were females (54%), with a median age of 35 years (IQR, 25-50 years). Of the 68 patients included, 61 were adult (median age, 37 years; IQR, 26-53 years), and 7 were pediatric (median age, 10 years; IQR, 4-11 years). Due to the low sample size, a subanalysis for pediatric patients was not carried out.

Fourteen different IPFD forms were represented (Supplementary Tables S3 and S4 and Supplementary Figure S1), among which there were GT (23.5%), PSD (14.7%), δ-granule deficiency (10.3%), biallelic BSS (bBSS) (7.3%), familial platelet disorder with predisposition to myeloid leukemia (7.3%), Gray platelet syndrome (7.3%), and others (29.6%).

Median baseline BS was 8 (IQR, 2.2-12) in the overall IPFD population, 8 (IQR, 2.5-12.5) in adult and 8 (IQR, 2-11) in pediatric patients; the BS was abnormal in 46 patients, 71% of the pediatric patients (5/7) and 67% of the adult patients (41/61). The median BS in this subpopulation did not statistically differ from the median BS of the entire BAT-VAL population (median, 9; IQR, 6-14; *n* = 198).

### 3.2 Association of platelet function assays with the BS

The results of 16 different laboratory assays (Supplementary Tables S2 and S5) were recorded in the case report form and here we show only those assays for which an adequate number of patients (≥15) was available for reliable statistical analysis.

### 3.3 LTA

Out of 67 patients with IPFD assessed by LTA, a platelet aggregation defect was found in 48 (71%).

The percentage of patients with high BS and defective LTA in response to ADP was significantly higher than that of patients with normal BS and defective LTA (59.4 ± 4.7% vs 35.5 ± 4.8%; *P* < .05).

**Figure 1:**
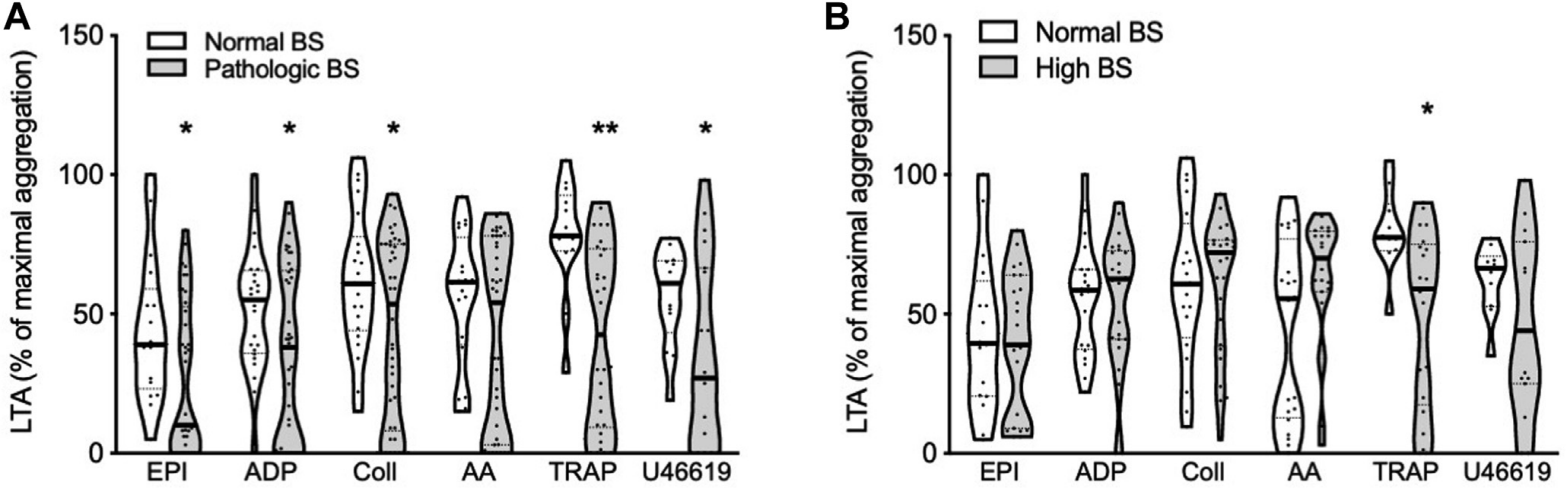
Percentage of maximal platelet aggregation as assessed by light transmission aggregometry (LTA) in response to different stimuli in patients with a normal or a high bleeding score (BS), including (A) all studied patients or (B) after the exclusion of patients with Glanzmann thrombasthenia and CalDAG-GEFI deficiency. Data are shown as violin plots, expressing median and quartiles, the frequency distribution curve, and the individual points (∗*P* < .05 vs normal BS; ∗∗*P* < .01 vs normal BS; 1-way analysis of variance). AA, arachidonic acid; Coll, collagen; EPI, epinephrine.

Maximal amplitude of platelet aggregation induced by all tested agonists (ADP, EPI, collagen, TRAP-6, and U46619), except arachidonic acid, was significantly lower in subjects with an abnormal BS (Figure 1A). The number of enrolled patients with bBSS was only 5, and all had defective responses to ristocetin (data not shown). An abnormal LTA in response to at least 2 agonists was significantly associated with higher BS (Supplementary Figure S2).

Moreover, a significant inverse correlation between maximal amplitude of aggregation and the ISTH-BS was found for ADP (r^2^ = 0.57; *P* < .001), EPI (r^2^ = 0.46, *P* < .001), collagen (r^2^ = 0.42, *P* < .001) and TRAP-6 (r^2^ = 0.52, *P* < .001) (Supplementary Figure S3).

Analysis performed by replacing the LTA response to the highest agonist dose with that to the lowest agonist dose, when available, showed that statistical significance was maintained and even strengthened (from *P* < .05 to *P* < .01 for LTA in response to ADP) (Supplementary Figure S4).

Among the single ISTH-BAT items, defective platelet aggregation in response to U46619 was associated with severe bleeding after surgery, while defective platelet aggregation in response to ADP and TRAP-6 with severe menorrhagia (Supplementary Table S6).

ROC curve analysis showed that a platelet aggregation defect predicted a pathologic BS with an AUC of 0.69 for ADP, 0.68 for collagen, 0.72 for EPI, and 0.87 for TRAP-6, showing good predictive ability in the latter case (Supplementary Figure S5).

Maximal amplitudes of aggregation of ≤17% for ADP (OR, 6.1; 95% CI, 1.7-21.1; *P* < .01), <48% for collagen (OR, 2.6; 95% CI, 1.01-6.9; *P* < .05), <14% for EPI (OR, 2.139; 95% CI, 0.744-6.151; *P* = .12), and <71% for TRAP-6 (OR, 3.6; 95% CI, 1.01-12.8; *P* < .05) were significantly associated with an increased probability of having an abnormal BS.

Excluding GT and CalDAG-GEFI deficiency cases, which are characterized by absence of or strongly reduced aggregation in response to all agonists, the association of enhanced BS with reduced percentage of maximal aggregation remained only for TRAP-6 (Figure 1B). This is probably in part due to the reduction of the power of the statistical test after the exclusion of GT and CalDAG-GEFI deficiency cases, which make up approximately one-third of the study patients. For instance, for aggregation induced by EPI, the power of the test dropped from 100% to 20.1% and by ADP from 100% to 6.3%. On the contrary, for aggregation induced by TRAP-6, the power of the test changed from 100% to only 99.8%.

### 3.4 BT and PFA-100

**Figure 2:**
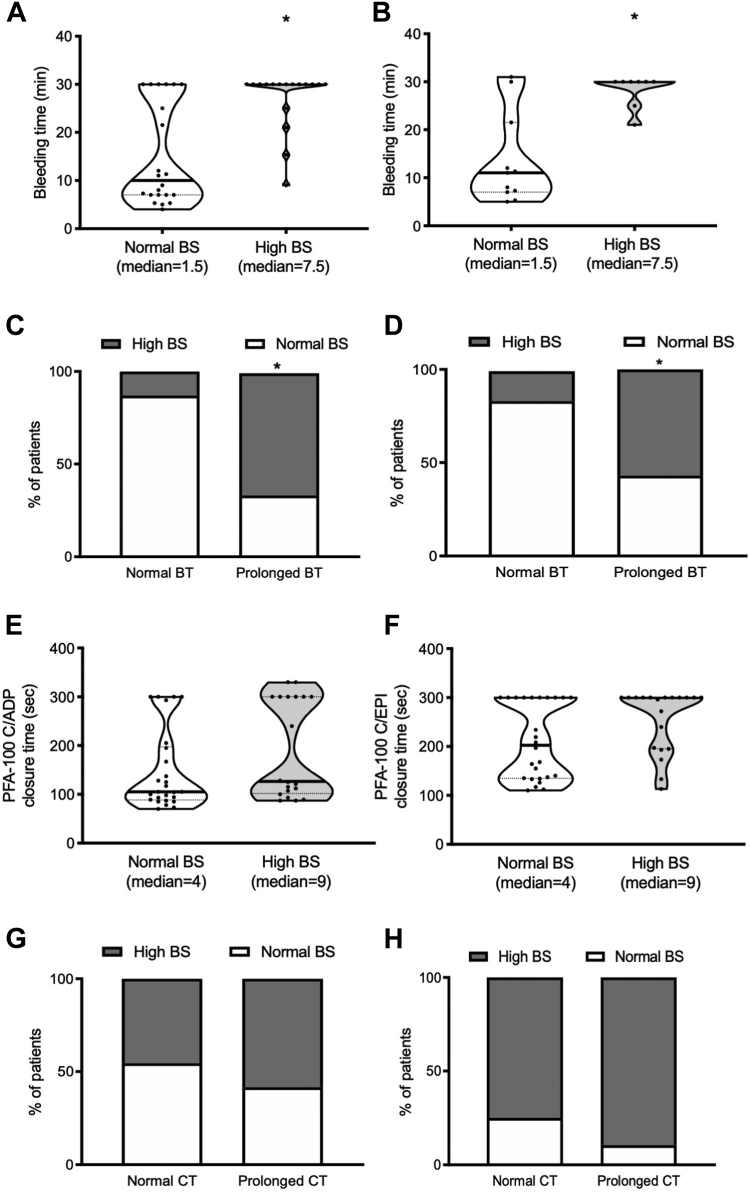
Bleeding time in patients with normal or high bleeding score (BS), including all (A) studied patients or (B) after the exclusion of patients with Glanzmann thrombasthenia and CalDAG-GEFI deficiency (∗*P* < .05 vs normal BS, Mann–Whitney U-test). Percentage of patients with normal or high BS according to normal or prolonged bleeding time, including (C) all studied patients or (D) after the exclusion of patients with Glanzmann thrombasthenia and CalDAG-GEFI deficiency (∗*P* < .05 vs normal BS, Mann–Whitney U-test). PFA-100 closure time (CT) with the (E) collagen (C)/ADP and (F) C/epinephrine (EPI) cartridges in patients with a normal or a high BS. Data are shown as violin plots, expressing median and quartiles, the frequency distribution curve, and the individual points. Percentage of patients with normal or high BS according to normal or prolonged PFA-100 CT with the (G) C/ADP or (H) C/EPI cartridges.

The BT and PFA-100 CT were altered in 64% and 88% of tested patients, respectively. The BT was significantly more prolonged in patients with a high BS than in those with a normal BS (Figure 2A). Excluding GT and CalDAG-GEFI deficiency cases, the association of pathologic BS with BT prolongation remained statistically significant (Figure 2B). An abnormal BT was significantly more common among patients with a pathologic BS than among those with a normal BS (Figure 2C), even after exclusion of GT and CalDAG-GEFI deficiency cases (Figure 2D). On the contrary, neither collagen/ADP nor collagen/EPI PFA-100 CTs were different between patients with normal and those with high BS (Figure 2E, F).

Moreover, a significant direct correlation between the BT and the ISTH-BAT BS was found (r = 0.72; *P* < .01) (Supplementary Figure S6), while no association was found between PFA-100 CT and the BS (Figure 2G, H).

ROC curve analysis revealed moderate discriminative ability of the BT for pathologic BS, with an AUC of 0.795 (*P* < .001), also after exclusion of patients with GT and CalDAG-GEFI deficiency. The best cutoff of BT for discriminating pathologic BS from normal BS was >12 minutes, with a sensitivity of 94%, specificity of 64%, positive predictive value of 64% (95% CI, 42.5-82.0), and a negative predictive value of 93% (66.1-99.8) (Supplementary Figure S7) (OR, 8.273; 95% CI, 0.87-78.01; *P* = .79).

ROC curve analysis showed no discriminative ability of PFA-100 for pathologic BS with an AUC of 0.642 (*P* = .06) (OR, 0.35; 95% CI, 0.05-2.24; *P* = .07).

### 3.5 Platelet surface GPIIb/IIIa expression and activation

GPIIb/IIIa surface expression by flow cytometry was abnormal in 27% of tested patients, and GPIIb/IIIa activation (PAC-1 binding) in 39%.

**Figure 3:**
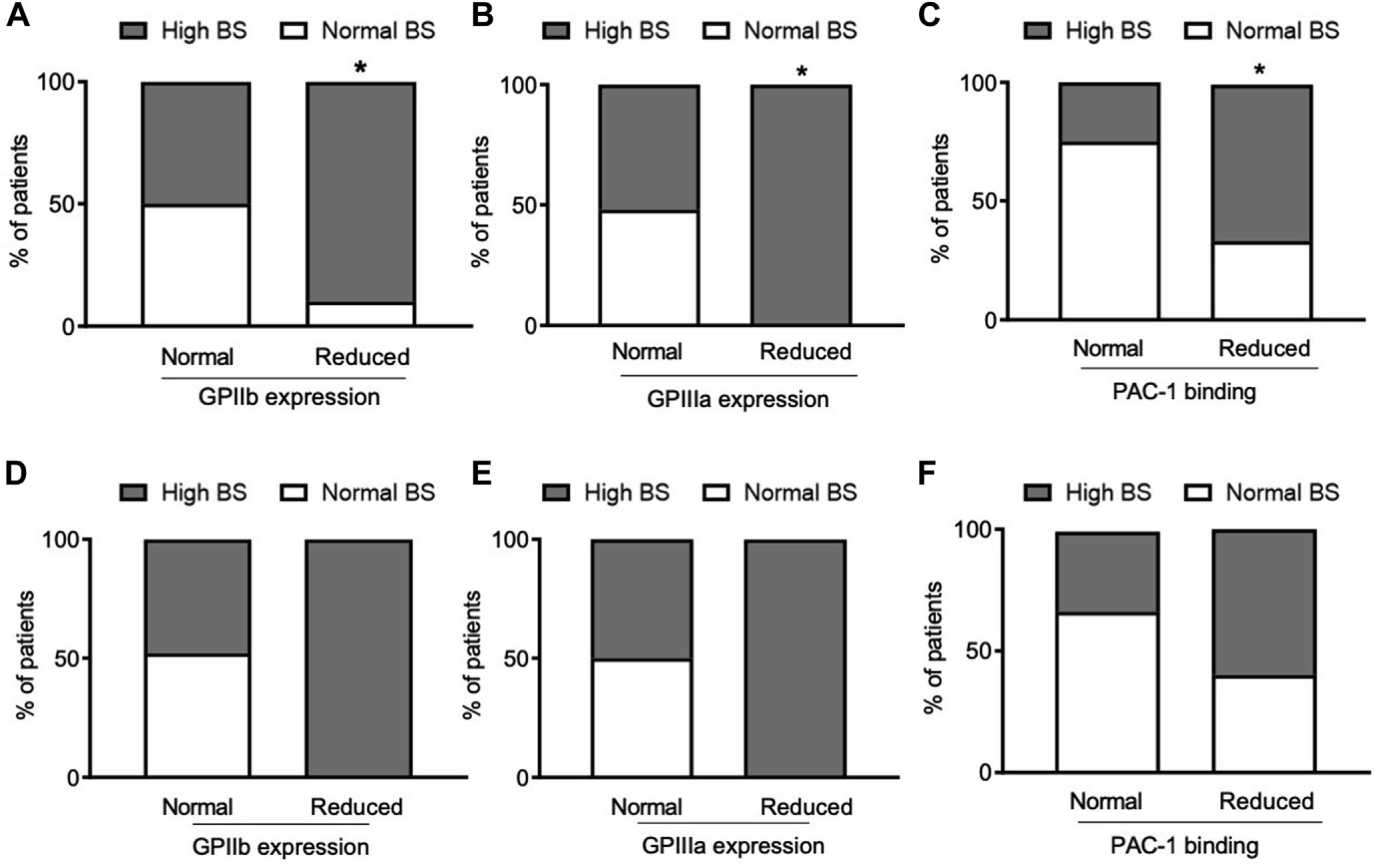
Percentage of patients with a normal or high bleeding score (BS) according to normal or defective expression of glycoprotein (GP) IIb, GPIIIa, or PAC-1 binding, as assessed by flow cytometry, including (A–C) all studied patients or (D–F) after the exclusion of patients with Glanzmann thrombasthenia and CalDAG-GEFI deficiency (∗*P* < .05 vs normal BS, chi-squared test).

Reduced GPIIb/IIIa expression was significantly more frequent in patients with a pathologic BS: only 9% of patients with defective GPIIb and 0% of patients with defective GPIIIa had a normal BS (Figure 3A, B) (GPIIb: OR, 7.2; 95% CI, 1.2-40.6; *P* < .05; GPIIIa: OR, 7.5; 95% CI, 0.759-74.157; *P* = .08).

Moreover, defective PAC-1 binding was more frequent in patients with a high BS (66%) than in patients with a normal BS (33%) (Figure 3C) (OR, 6.2; 95% CI, 1.2-32.2; *P* < .05; 31% were GT).

Among the single ISTH-BAT items, defective PAC-1 binding upon activation by ADP was associated with severe bleeding after tooth extraction (Supplementary Table S6).

ROC curve analysis revealed moderate discriminative ability of PAC-1 binding for BS with an AUC of 0.778 (*P* < .001). The best PAC-1 binding cutoff for discriminating pathologic BS from normal BS was <3.7 mean fluorescence intensity (Supplementary Figure S8).

Excluding GT and CalDAG-GEFI deficiency cases, the associations of pathologic BS with reduced GPIIb/IIIa expression and impaired PAC-1 binding were no longer significant (Figure 3D–F).

### 3.6 α/δ-Granules content and release

**Figure 4:**
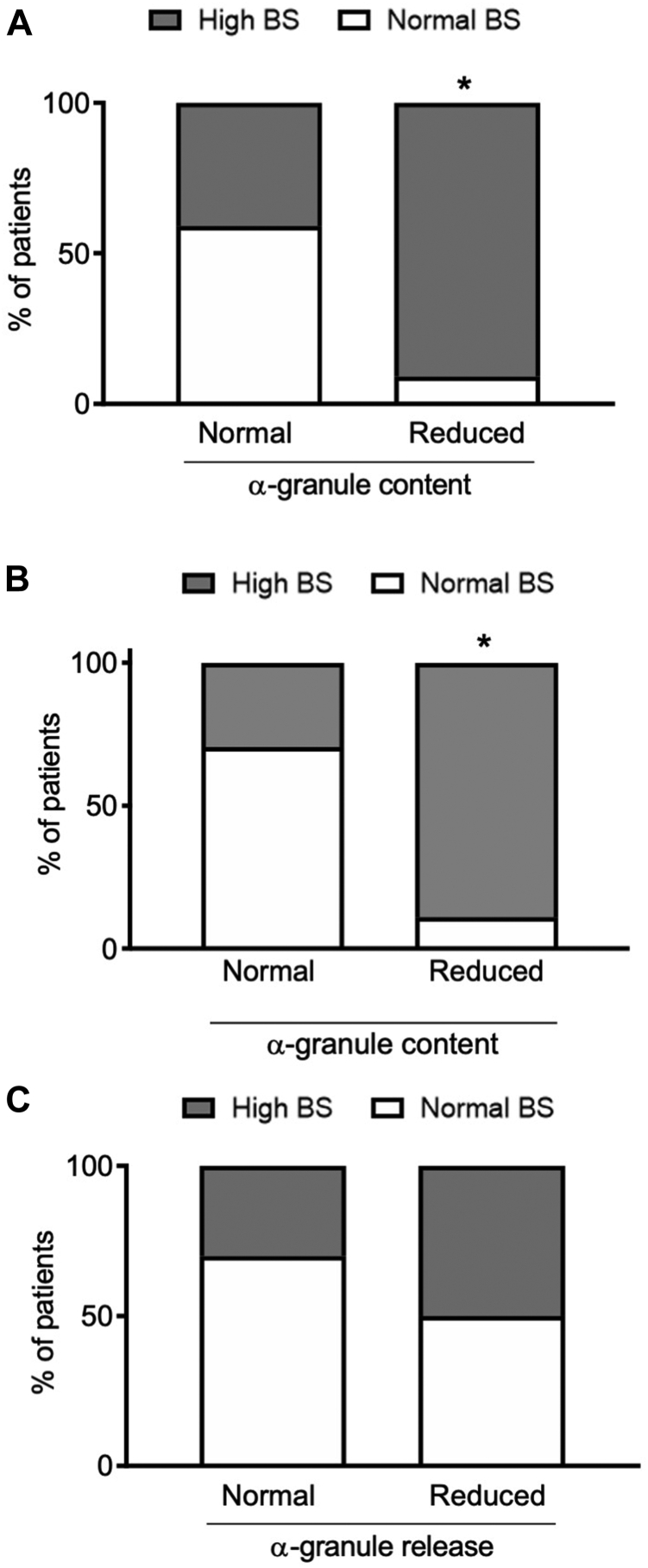
Percentage of patients with a normal or high bleeding score (BS) according to normal or defective α-granule content, including (A) all studied patients or (B) after the exclusion of patients with Glanzmann thrombasthenia and CalDAG-GEFI deficiency or normal or defective α-granule release (C) (∗*P* < .05 vs normal BS, chi-squared test).

Reduced α-granule content was found in 29% of tested patients, and it was significantly more common among those with a pathologic BS (Figure 4A) (OR, 9; 95% CI, 1.5-50.6; *P* < .05). Excluding GT and CalDAG-GEFI deficiency cases, the association of pathologic BS with reduced α-granule content remained statistically significant (Figure 4B).

Instead, no correlation was found between α-granule release and the BS (OR, 7.2; 95% CI, 0.410-43.035; *P* = .22) (Figure 4C). Among the single ISTH-BAT items, defective content of α-granules was associated with severe epistaxis and bleeding after surgery (Supplementary Table S6).

On the contrary, dense-granule content or release was not correlated with the BS (content: OR, 0.857 95% CI, 0.20721-3.552; *P* = .81; release: OR, 1; 95% CI, 0.212-4.709).

Patients with concomitant defective α-granule content and aggregation (at least 1 agonist) had a higher BS than that of patients with only 1 defective test (Supplementary Figure S9). All the results described above were comparable to those obtained by analyzing data after exclusion of the pediatric population.

### 3.7 Other tests

Platelet count and MPV were not different between patients with a normal BS and patients with a high BS; moreover, we did not find any correlation between laboratory assay results and these parameters.

Finally, there was no association between clot retraction or serum TxB_2_ and the ISTH BS. Platelet electron microscopy (TEM) was performed in only 8 subjects; therefore, results were inconclusive.

## 4 Discussion

In the current study, we examined whether the degree of impairment of platelet function laboratory assays is associated with the severity of bleeding in patients with IPFD. The ISTH-BAT has been previously validated cross-sectionally and prospectively to assess the severity of bleeding diathesis in patients with an IPFD, showing that it reflects the severity of patient bleeding tendency [7,9].

Our data suggest that abnormal platelet function laboratory assay results are associated with a more severe bleeding tendency in IPFD. However, we could not identify a single test defective in all patients with an abnormal BS; for instance, 5 of 46 patients (11%) with a pathologic BS had completely normal LTA but an impaired granule content or release, confirming that platelet secretion studies must be performed for IPFD diagnostics even when LTA is normal [20,28]. This suggests that a single test may not be sufficient to predict an increased BS but that a panel of laboratory assays is required. This confirms previous data showing that LTA is valuable for detecting platelet function abnormalities, particularly when the test shows abnormal responses to multiple agonists [29]. Indeed, in our study, an abnormal LTA to at least 2 agonists was associated with a higher BS with particular strength.

In particular, we found that impaired LTA was associated with a high likelihood of an abnormal BS. These findings are in agreement with previously published results showing the same association in patients with GT, bBSS, defective aggregation in response to adrenaline, dense granule deficiency, familial platelet disorder with predisposition to myeloid leukemia [16,18], or a generic diagnosis of platelet function disorder [19]. As expected, after the exclusion of patients with GT and CalDAG-GEFI deficiency, who notoriously display a severe platelet aggregation defect, the association of a pathologic BS with reduced aggregation remained only for TRAP-6. This may be due in part to the relatively low statistical power remaining after the exclusion of one-third of the enrolled patients. Concerning the finding of a significant association between lower TRAP-6-induced LTA and a pathologic BS, this is in line with previous data showing an association between bleeding severity and impaired platelet activation through TRAP-6 in patients with immune thrombocytopenia [30].

Moreover, we showed an association between impaired GPIIb/IIIa expression and activation as well as reduced α-granule content with increased BS, which however was no longer significant after the exclusion of patients with GT and CalDAG-GEFI deficiency. In addition, we found that prolongation of BT correlated with an abnormal BS, confirming that, even if not recommended because of insufficient specificity, sensitivity, and/or low reproducibility [20], this may be a potentially useful optional assay for suspected IPFD when performed by skilled operators [31]. BT prolongation and reduced α-granule content remained significantly associated with BS even after the exclusion of patients with GT and CalDAG-GEFI deficiency.

We also found an association between some specific items of the ISTH-BAT and platelet function assay results, thus corroborating a correlation between platelet function assays and the main typical bleeding manifestations of mucocutaneous bleeding disorders.

We did not find any correlation instead between dense-granule content and an abnormal BS, which is not in line with the findings of other authors [18,19]; however, they assessed dense-granule content by TEM, while in our study, TEM was performed only in a small number of subjects, making the results inconclusive. Indeed, according to our results, dense-granule content assessed by lumiaggregometry did not correlate with bleeding manifestations, as in some previous studies [14,15]. We also did not find any correlation between PFA-100 and an abnormal BS, while in a previous study, a prolonged collagen/EPI PFA-100 CT was significantly associated with the bleeding severity in patients with clinical suspicion of bleeding disorder; however, in that study, only 9% of the study population was represented by patients with a platelet defect [32].

Our results differ from some previous studies, which did not identify a correlation between defective platelet aggregation [1,14,15], platelet GP expression [15], α-granule content [15], and the bleeding diathesis. However, one study included subjects without a conclusive diagnosis of IPFD [1], another excluded patients with GT, BSS, MYH9-related disorder, and Hermansky-Pudlak Syndrome [14], and the third investigated only patients with PSDs [15], limiting their generalizability.

In our case series, some subjects affected by IPFD, conventionally considered as severe, showed a normal ISTH-BAT BS. We do not have an explanation for this, but it has been reported that among patients with some usually severe forms, the ISTH-BAT BS may sometimes be only mildly altered or normal, reflecting the phenotypic heterogeneity of IPFD [3,7]. Even the spectrum of clinical bleeding manifestations of patients with GT, one of the most severe bleeding disorders, has been reported to range from severe/fatal to rather mild [33].

Strengths of our study are the inclusion of several laboratory assays, the enrollment of patients with a well-defined IPFD diagnosis and a wide range of forms, and the worldwide representation of participating centers.

Limitations of our study are: 1) the relatively small number of enrolled patients; however, these were very well characterized; 2) the inclusion of a substantial number of severe disorders, such as BSS and GT, which, however, were present in a similar or even higher percentage in 2 previous large IPFD case series [7,8]; 3) the relatively low number of records for some tests, which however are carried out only by a few specialized laboratories worldwide [34]; 4) the fact that no patients were included for whom all the tests were available; 5) the fact that laboratory tests were not centralized; and 6) the post hoc nature of the study and, thus, the lack of a prospective design. Moreover, we did not collect information on the race/ethnicity of participants, and we acknowledge this as a limitation because we might have missed information on the socio-cultural determinants of bleeding severity of the studied population and because a racial difference has been described for PAR4-mediated platelet aggregation [35].

Although we acknowledge the limitations of this study, we believe that our study suggests that a diagnostic approach integrating the assessment of the ISTH-BAT BS with the results of standardized laboratory platelet function assays may help to more precisely define the future bleeding risk of patients with IPFD.

In conclusion, we found that some platelet function laboratory assays may predict an enhanced bleeding tendency in patients with IPFD, with important implications for the management of these patients. A prospective validation of the results of this study by a larger, prospective, international collaborative study is highly warranted.
