# Supplementary material for: Association of laboratory test results with the bleeding history in patients with inherited platelet function disorders (the Bleeding Assesment Tool - LABoratory tests substudy): communication from the Platelet Physiology ISTH-SSC
Source: Res Pract Thromb Haemost. 2023 Dec 20;8(1):102305. doi: 10.1016/j.rpth.2023.102305 (PMC10825541; doi:10.1016/j.rpth.2023.102305)
Supplement: Supplementary material [file mmc1.docx]

**Supplementary material**

**Case Report Form**

| Patient ID (same of BAT evaluation study): |  |
| --- | --- |
| Date of enrolment: |  |
| ISTH-BAT bleeding score: |  |

- **Platelet count:**

| **Platelet count** | **Number (x10^9^/L)** |
| --- | --- |
| Counter |  |
| Microscope |  |

- **Platelet size:**

| **Mean platelet volume** | **fL** | **reference intervals**  **of the laboratory** |
| --- | --- | --- |
| MPV |  |  |

| **Mean platelet diameter** | **μm** | **reference intervals**  **of the laboratory** |
| --- | --- | --- |
| MPD |  |  |

| **Blood smear** | **%** | **Presence/absence** |
| --- | --- | --- |
| small |  |  |
| normal |  |  |
| large |  |  |

- **Platelet morphology:**

| **Blood smear** | **Presence/absence** |
| --- | --- |
| “grey” or “pale” platelets |  |
| Others |  |
|  |  |
| **Transmission Electron Microscopy** | **Type of abnormality** |
| Granule morphology |  |
| Other structural abnormalities |  |

- **Other cells morphology:**

| **Blood smear** | **Presence/absence** |
| --- | --- |
| Howell-Jolly bodies in red blood cells |  |
| Abnormal giant granules in eosinophils, basophils and monocytes |  |
| Dyserytropoiesis |  |
| Leukocyte inclusion bodies |  |
| Other (please describe) |  |

- **Light Transmission Aggregometry:**

| **Platelet agonist** | **Concentration** | **Maximal amplitude**  **of aggregation (%)** | **Reversible**  **Aggregation (yes/no)** | **reference intervals**  **of the laboratory** |
| --- | --- | --- | --- | --- |
| Epinephrine |  |  |  |  |
| ADP |  |  |  |  |
| Collagen |  |  |  |  |
| Arachidonic Acid |  |  |  |  |
| α-thrombin |  |  |  |  |
| TRAP-6 |  |  |  |  |
| U46619 |  |  |  |  |
| CRP |  |  |  |  |
| CVX |  |  |  |  |
| PAR4-ap |  |  |  |  |
| PAF |  |  |  |  |
| A23187 |  |  |  |  |
| Ristocetin |  |  |  |  |
| RIPA (minimum dose of ristocetin inducing platelet agglutination) |  |  |  |  |

- **Impedance aggregometry (Multiplate):**

| **Platelet agonist** | **Concentration** | **Aggregation (AUC)** | **Velocity (AUC/min)** | **reference intervals**  **of the laboratory** |
| --- | --- | --- | --- | --- |
| TRAP-6 |  |  |  |  |
| ADP |  |  |  |  |
| Collagen |  |  |  |  |
| Arachidonic Acid |  |  |  |  |

- **Granules release:**

| **α granules** | **Kind of analyte** | **Normal/defective** |
| --- | --- | --- |
| ELISA |  |  |
| Flow cytometry |  |  |
|  |  |  |
| **δ granules** | **Kind of analyte** | **Normal/defective** |
| Lumiaggregometry |  |  |
| Luminometry |  |  |
| HPLC |  |  |
| Flow cytometry |  |  |

- **Granules content:**

| **α granules** | **Kind of analyte** | **Normal/defective** |
| --- | --- | --- |
| ELISA |  |  |
| Flow cytometry |  |  |
| Immunofluorescence |  |  |
|  |  |  |
| **δ granules** | **Kind of analyte** | **Normal/defective** |
| Lumiaggregometry |  |  |
| Luminometry |  |  |
| HPLC |  |  |
| TEM |  |  |
| Flow cytometry |  |  |

- **Flow cytometry:**

| **Surface antigens:** | **% of positive cells** | **MFI** | **reference intervals**  **of the laboratory** |
| --- | --- | --- | --- |
| GPIIb |  |  |  |
| GPIIIa |  |  |  |
| GPIX |  |  |  |
| GPIbalpha |  |  |  |
| GPIa/IIa |  |  |  |
| GPIV |  |  |  |
| GPVI |  |  |  |
| Others |  |  |  |

| **Markers of platelet activation** | **Kind**  **of agonist** | **Dose**  **of agonist** | **% of positive**  **cells** | **MFI** | **reference intervals of the laboratory** |
| --- | --- | --- | --- | --- | --- |
| GPIIb/IIIa activation  (PAC-1) |  |  |  |  |  |
| surface expression of CD62P (α−granules integral membrane protein P-selectin) |  |  |  |  |  |
| surface expression of CD63 (Lysosomes and dense bodies integral membrane protein LIMP-1) |  |  |  |  |  |
| Platelet procoagulant activity (annexin V binding) |  |  |  |  |  |

- **Serum TxB2:**

|  | **ng/ml** | **ng/10^8^ platelets** | **reference intervals of the laboratory** |
| --- | --- | --- | --- |
| Serum TxB2 |  |  |  |

- **Clot retraction:**

|  | **Normal/defective** |
| --- | --- |
| Clot retraction |  |

- **Mixing tests (LTA):**

|  | **Enhanced/normal** |
| --- | --- |
| Patient’s plasma + control plts |  |
| Control’s plasma + patient plts |  |

- **Mixing tests (Flow-cytometry):**

|  | **Enhanced/normal** |
| --- | --- |
| Patient’s plasma + control plts |  |
| Control’s plasma + patient plts |  |

- **Molecular genetic diagnosis:**

|  | **Mutation** |
| --- | --- |
| Gene |  |

- **Additional tests:**

|  | **Method** | **Minutes** | **reference intervals of the laboratory** |
| --- | --- | --- | --- |
| Bleeding time assay |  |  |  |

| **PFA-100 test** | **Seconds** | **reference intervals of the laboratory** |
| --- | --- | --- |
| CADP |  |  |
| CEPI |  |  |

- **OTHER TESTS**

| **Test** | **Results** | **reference intervals of the laboratory** |
| --- | --- | --- |
|  |  |  |

| **Supplementary table 1**. Diagnostic criteria for IPFD patients enrolled in the study. | | | |
| --- | --- | --- | --- |
| ***Disease (abbreviation, OMIM entry)*** | ***Inheritance*** | ***Gene (chromosome localization)*** | **Diagnostic criteria** |
| *Hermansky–Pudlak syndrome (HPS, 203300)* | AR | *HPS1, ADTB3A, HPS3, HPS4, HPS5,HPS6, DTNBP1, BLOC1S3* BLOC1S3 (different locations) HPS4, HPS5,HPS6, DTNBP1 BLOC1S3 (different locations) | Genetic analysis or typical phenotype + delta granule deficiency or decrease in platelet nucleotide content and increased ATP/ADP ratio (+decreased 5HT content) |
| *Glanzmann thrombasthenia (GT, 273800)* | AR | *ITGA2B* (17q21.31),  *ITGB3* (17q21.32) | Genetic analysis or absent platelet aggregation to all agonists but ristocetin or absent GPIIb-IIIa |
| ^§^*ITGA2B*/*ITGB3*-related thrombocytopenia (*ITGA2B*/*ITGB3-RT*, 187800) | AD | *ITGA2B* (17q21.31),  *ITGB3* (17q21.32) | Genetic analysis |
| ^§^Biallelic Bernard Soulier Syndrome (bBSS, 231200) | AR | *GP1BA* (17p13), *GP1BB* (22q11), *GP9* (3q21) | Genetic analysis or absent GPIb/IX/V or absent RIPA and normal to other agonists |
| ^§^Familial platelet disorder and predisposition to acute myelogenous leukemia (FPD/AML, 601399) | AD | *RUNX1* (21q22) | Genetic analysis |
| *P2Y_12_ deficiency (nd, 609821)* | AR | *P2RY12* (3q24-q25) | Genetic analysis or selective, severe defect of platelet aggregation by ADP, defect of inhibition o adenylyl cyclase by ADP (VASP phosphorylation assay) |
| *Defect of thromboxane A2 receptor (nd, 188070)* | AD | *TBXA2R* (19p13.3) | Genetic analysis or defective platelet aggregation by U46619 and by arachidonic acid |
| *Delta storage pool disease (nd, nd)* | AR/AD | *Unknown* | Absence of delta-granules (TEM) or decrease in platelet nucleotide content and increased ATP/ADP ratio (+decreased 5HT content) |
| *Combined alpha-delta granule deficiency (nd, 185050)* | AR/AD | Unknown | Severe deficiency of alpha and delta granules |
| ^§^*Platelet-type Von Willebrand Disease (VWDP, 177820)* | AD | *GP1BA (17p13.2)* | Genetic analysis |
| ^§^*Gray platelet syndrome (GPS, 139090)* | AR | *NBEAL2* (3p21.1) | Genetic analysis or absence of alpha-granules |
| *CalDAG_GEFI defect (nd,* 615888) | AR | *RASGRP2* (11q13.1) | Genetic analysis |
| *Primary secretion defect (nd, nd)* | AR/AD | *Unknown* | Reduced primary platelet granule secretion upon stimulation by different platelet aggregation agonists, normal TxB2 production induced by AA (or serum TxB2) and normal granule content. |
| *Defects in collagen receptors (nd, nd)* | AR | *Unknown* | Defective platelet aggregation in the response to collagen |
| *cPLA2 deficiency* | *AR* | *PLA2G4A (1q31.1)* | *Genetic analysis* |

**Supplementary table 2**

**Platelet Count**

|  | Instrument name (company) | normal range (x10^9^/L) |
| --- | --- | --- |
| Perugia | Emerald Cell-Dyn (Abbott) | 150-400 |
| Marseille | Advia 120 (Siemens) | 150-400 |
| Milan | XN1000 (Dasit) | 150-300 |
| Buenos Aires | ACT diffTM (Coulter Corp) | 150-400 |
| Rome | ADVIA 2120i (Siemens) | 150-450 |
| Pessac | DxH 500 (Beckman Coulter) | 150-400 |
| Philadelphia | CN6000 (Sysmex) | 150-450 |
| Caceres | Sysmex (Roche) | 150-400 |
| Coimbra | Cell Dyn Sapphire (Abott) | 150-400 |
| Detroit | XM 2000 (Sysmex) | 130 - 450 |
| Bergamo | NE800 Analyzer (Dasit) | 150 - 400 |

**MPV**

|  | Instrument name (company) | normal range (fL) |
| --- | --- | --- |
| Perugia | Emerald Cell-Dyn (Abbott) | 7-11 |
| Marseille | Advia 120 (Siemens) | 7-9 |
| Milan | XN1000 (Dasit) | 9.3-12.6 |
| Buenos Aires | ACT diffTM (Coulter Corp) | 6.2-10.4 |
| Rome | ADVIA 2120i (Siemens) | 6.8-10 |
| Pessac | DxH 500 (Beckman Coulter) | 7-10 |
| Philadelphia | CN 6000 (Sysmex) | 9.2-11.4 |
| Caceres | Sysmex (Roche) | 6-12 |
| Coimbra | Cell Dyn Sapphire (Abott) | 8.17 - 9.65 |
| Detroit | XM 2000 (Sysmex) | 7.3-11.4 |
| Bergamo | NE800 Analyzer (Dasit) | 7.2-12 |

**PFA-100® (C/EPI)**

|  | Instrument name (company) | normal range (sec) |
| --- | --- | --- |
| Perugia | PFA-100 (Siemens Healthcare Diagnostic) | 82-150 |
| Marseille | PFA-100 (Siemens Healthcare Diagnostic) | 82-165 |
| Milan | PFA-100 (Siemens Healthcare Diagnostic) | 76-178 |
| Buenos Aires | not performed | not performed |
| Rome | PFA-100 (Siemens Healthcare Diagnostic) | <140 |
| Pessac | PFA-100 (Siemens Healthcare Diagnostic) | <145 |
| Philadelphia | not performed | not performed |
| Caceres | PFA-100 (Siemens Healthcare Diagnostic) | 110-160 |
| Coimbra | PFA-100 (Siemens Healthcare Diagnostic) | 91-155 |
| Detroit | PFA-100 (Siemens Healthcare Diagnostic) | 100 - 163 |
| Bergamo | PFA-100 (Siemens Healthcare Diagnostic) | <188 |

**PFA-100® (C/ADP)**

|  | Instrument name (company) | normal range (sec) |
| --- | --- | --- |
| Perugia | PFA-100 (Siemens Healthcare Diagnostic) | 62-100 |
| Marseille | PFA-100 (Siemens Healthcare Diagnostic) | 62-128 |
| Milan | PFA-100 (Siemens Healthcare Diagnostic) | 60-116 |
| Buenos Aires | not performed | not performed |
| Rome | PFA-100 (Siemens Healthcare Diagnostic) | <120 |
| Pessac | PFA-100 (Siemens Healthcare Diagnostic) | <110 |
| Philadelphia | not performed | not performed |
| Caceres | PFA-100 (Siemens Healthcare Diagnostic) | 85-120 |
| Coimbra | PFA-100 (Siemens Healthcare Diagnostic) | 67-99 |
| Detroit | PFA-100 (Siemens Healthcare Diagnostic) | 57 - 114 |
| Bergamo | PFA-100 (Siemens Healthcare Diagnostic) | <135 |

**Bleeding Time**

|  | Method | normal range (minutes) |
| --- | --- | --- |
| Perugia | Mielke | 4-7 |
| Marseille | Ivy | 4-8 |
| Milan | not performed | not performed |
| Buenos Aires | not performed | not performed |
| Rome | Ivy | <8 |
| Pessac | not performed | not performed |
| Philadelphia | not performed | not performed |
| Caceres | not performed | not performed |
| Coimbra | not performed | not performed |
| Detroit | not performed | not performed |
| Bergamo | not performed | not performed |

**Aggregometry with ADP**

|  | Type | Instrument name (company) | Dose of the agonist (μM) | normal range |
| --- | --- | --- | --- | --- |
| Perugia | LTA | APACT 4 (Helena) | 10 | 59.8±12.4 (43.2-73.3) |
| Marseille | LTA | APACT 4 (Helena) | 2.5  10 | 75-95  80-95 |
| Milan | LTA | Chronolog 400VS (Mascia Brunelli) | 2/4/20 | >39% |
| Buenos Aires | LTA | Chronolog V500 (Mascia Brunelli) | 2/10 | 75±9.8% |
| Rome | LTA | AggRAM (Helena) | 2/4/10 | >50/>70%/>70% |
| Pessac | LTA | APACT 4 (Helena) | 10 | >65 |
| Philadelphia | Impedence | Chronolog 700 (Mascia Brunelli) | 10 | >6 ohms |
| Caceres | LTA | Chronolog 700 (Mascia Brunelli) | 1  2  4 | 55±27%  70±20%  90±12% |
| Coimbra | LTA | 490-4D Optic Aggregometer (Chronolog) | 10  5 | 71-115  69-118% |
| Detroit | LTA | Chronolog 700 (Mascia Brunelli) | 10 | 51 – 98% |
| Bergamo | LTA | Chronolog 700 (Mascia Brunelli) | 0.8 | double wave of aggregation |

**Aggregometry with epinephrine**

|  | Type | Instrument name (company) | Dose of the agonist (μM) | normal range |
| --- | --- | --- | --- | --- |
| Perugia | LTA | APACT 4 (Helena) | 100 and 10 | 56.9±12.8 (44.1-69.7) |
| Marseille | LTA | APACT 4 (Helena) | 10 | 74-87 |
| Milan | LTA | Chronolog 400VS (Mascia Brunelli) | 5 | > 42% |
| Buenos Aires | LTA | Chronolog V500 (Mascia Brunelli) | 1 | 78±9.5% |
| Rome | LTA | AggRAM (Helena) | 5 and 25 | >70% |
| Pessac | LTA | APACT 4 (Helena) | 4 | >70 |
| Philadelphia | not performed | not performed | not performed | not performed |
| Caceres | LTA | Chronolog 700 (Mascia Brunelli) | 10 | 75+25% |
| Coimbra | LTA | 490-4D Optic Aggregometer (Chronolog) | 10  5 | 45-142%  28-121% |
| Detroit | LTA | Chronolog 700 (Mascia Brunelli) | 5 | 69 – 88% |
| Bergamo | LTA | Chronolog 700 (Mascia Brunelli) | 1 | double wave of aggregation |

**Aggregometry with collagen**

|  | Type | Instrument name (company) | Dose of the agonist (μg/ml) | normal range |
| --- | --- | --- | --- | --- |
| Perugia | LTA | APACT 4 (Helena) | 2 | 69.0±11.2 (57.8-80.2) |
| Marseille | LTA | APACT 4 (Helena) | 2  10 | 78-90  78-90 |
| Milan | LTA | Chronolog 400VS (Mascia Brunelli) | 2  4  10 | > 64% |
| Buenos Aires | LTA | Chronolog V500 (Mascia Brunelli) | 2/4 | 77±11.8 % |
| Rome | LTA | AggRAM (Helena) | 2/4/5/10 | >70% |
| Pessac | LTA | APACT 4 (Helena) | 2 | >73 |
| Philadelphia | Impedence | Chronolog 700 (Mascia Brunelli) | 1/5 | >9 ohms |
| Caceres | LTA | Chronolog 700 (Mascia Brunelli) | 2.5 | 90+15% |
| Coimbra | LTA | 490-4D Optic Aggregometer (Chronolog) | 2 | 60-134% |
| Detroit | LTA | Chronolog 700 (Mascia Brunelli) | 2  5 | 64 – 86%  61 – 89% |
| Bergamo | LTA | Chronolog 700 (Mascia Brunelli) | 2 | irreversible aggregation |

**Aggregometry with TRAP-6**

|  | Type | Instrument name (company) | Dose of the agonist (μM) | normal range |
| --- | --- | --- | --- | --- |
| Perugia | LTA | APACT 4 (Helena) | 10 | 72.3±10.3 (62-82.6) |
| Marseille | LTA | APACT 4 (Helena) | 50 | 82-91 |
| Milan | LTA | Chronolog 400VS (Mascia Brunelli) | 10  20 | >38% |
| Buenos Aires | LTA | Chronolog V500 (Mascia Brunelli) | 20 | comparison vs. normal control assayed simultaneously |
| Rome | LTA | AggRAM (Helena) | 25-40-80 | >70% |
| Pessac | LTA | APACT 4 (Helena) | 25 | >74 |
| Philadelphia | not performed | not performed | not performed | not performed |
| Caceres | not performed | not performed | not performed | not performed |
| Coimbra | LTA | 490-4D Optic Aggregometer (Chronolog) | 25 | 81-113% |
| Detroit | LTA | Chronolog 700 (Mascia Brunelli) | 10 | 78 – 100% |
| Bergamo | not performed | not performed | not performed | not performed |

**Aggregometry with arachidonic acid**

|  | Type | Instrument name (company) | Dose of the agonist (mM) | normal range |
| --- | --- | --- | --- | --- |
| Perugia | LTA | APACT 4 (Helena) | 1 | 69.2±11.1 (58.1-80.3) |
| Marseille | LTA | APACT 4 (Helena) | 1 | 79-94 |
| Milan | LTA | Chronolog 400VS (Mascia Brunelli) | 1 | >62% |
| Buenos Aires | LTA | ChronoLog V500 (Mascia Brunelli) | 1 | 79±9 % |
| Rome | LTA | AggRAM (Helena) | 1/1.5 | >70% |
| Pessac | LTA | APACT 4 (Helena) | 1 | >73 |
| Philadelphia | not performed | not performed | not performed | not performed |
| Caceres | LTA | Chronolog 700 (Mascia Brunelli) | 1.6 | 90+20% |
| Coimbra | LTA | 490-4D Optic Aggregometer (Chronolog) | 1.6 | 60-132% |
| Detroit | LTA | Chronolog 700 (Mascia Brunelli) | 0.5 | 64 – 99% |
| Bergamo | not performed | not performed | not performed | not performed |

**Aggregometry with U46619**

|  | Type | Instrument name (company) | Dose of the agonist (μM) | normal range |
| --- | --- | --- | --- | --- |
| Perugia | LTA | APACT 4 (Helena) | 1 | 71.2±15.5 (55.6-86.7) |
| Marseille | not performed | not performed | not performed | not performed |
| Milan | LTA | Chronolog 400VS (Mascia Brunelli) | 1 | >63% |
| Buenos Aires | not performed | not performed | not performed | not performed |
| Rome | not performed | not performed | not performed | not performed |
| Pessac | LTA | APACT 4 (Helena) | 5 | >73% |
| Philadelphia | not performed | not performed | not performed | not performed |
| Caceres | not performed | not performed | not performed | not performed |
| Coimbra | not performed | not performed | not performed | not performed |
| Detroit | not performed | not performed | not performed | not performed |
| Bergamo | not performed | not performed | not performed | not performed |

**α-Granule content**

|  | Method (kind of analyte) | Instrument | normal range |
| --- | --- | --- | --- |
| Perugia | ELISA (β-TG) | N/A | 2940-3527 ng/10^8^ plts |
| Marseille | ELISA (PAI1)  Electonic microscopy | N/A | 0.33-1.07µg/10^9^ plt |
| Milan | not performed | not performed | not performed |
| Buenos Aires | Immunofluorescence (Anti-thrombospondin) | N/A | 94.8-99.7 % platelets harbouring >5 granules TSP1+ |
| Rome | Immunofluorescence (Anti-thrombospondin) | N/A |  |
| Pessac | not performed | not performed | not performed |
| Philadelphia | not performed | not performed | not performed |
| Caceres | not performed | not performed | not performed |
| Coimbra | not performed | not performed | not performed |
| Detroit | not performed | not performed | not performed |
| Bergamo | not performed | not performed | not performed |

**δ-Granule content**

|  | Method (kind of analyte) | Instrument name (company) | normal range |
| --- | --- | --- | --- |
| Perugia | Lumiaggregometry (ATP) | Chronolog 700 (Mascia Brunelli) | 1.82-2.94 nmoli/10^8^ plt |
| Marseille | HPLC (serotonin)  Counting (fluorescence)  Whole mount | ThermoFisher | 0.51-1.31µg/10^9^ plt |
| Milan | Lumiaggregometry (ATP/ADP) | Chronolog 400VS (Mascia Brunelli) | ADP: 1.99 – 5.01 nmoli/10^8^ plt  ATP: 5.01 – 10.42 nmoli/10^8^ plt |
| Buenos Aires | not performed | not performed | not performed |
| Rome | not performed | not performed | not performed |
| Pessac | not performed | not performed | not performed |
| Philadelphia | Lumiaggregometry (ATP) | Chronolog (Mascia Brunelli) | 0.39-99 nmol/10^8^/plt |
| Caceres | not performed | not performed | not performed |
| Coimbra | not performed | not performed | not performed |
| Detroit | not performed | not performed | not performed |
| Bergamo | not performed | not performed | not performed |

**α-granule release**

|  | Method (kind of analyte) | Instrument name (company) | agonist (dose) | normal range |
| --- | --- | --- | --- | --- |
| Perugia | ELISA (β-TG) | N/A | ADP 5 µM  EPI 10 µM  AA 1 mM  TRAP 10 µM  Coll 2 µg/ml | 2013-2554  2310-2860  2276-2792  2263-2942  2590-3202  nmoli/10^8^ plt |
|  | flow-cytometry (P-selectin) | FC500 (Beckman Coulter) | ADP 10 µM  TRAP 20 µM | 41.9-73.4%; 2.6-6.2 (MFI)  50-93%; 3.5-8.9 (MFI) |
| Marseille | Flow cytometry  (P-Selectin) | FC500 (Beckman Coulter) | ADP 10µM  TRAP 50µM | 3.2-15.7 (MFI)  Ratio>4  5.6-12 (MFI)  Ratio>7 |
| Milan | not performed | not performed | not performed | not performed |
| Buenos Aires | flow-cytometry (P-selectin) | FACS Calibur (Becton Dickinson) | ADP 10uM TRAP-6 10uM & 20uM | <80% vs normal control platelets (MFI) |
| Rome | flow-cytometry (P-selectin) | FC500 (Beckman Coulter) | PAR1-AP 25μM  ADP 10μM | Abnormal if MFI <50% of control value |
| Pessac | flow-cytometry (P-selectin) | FC500 (Beckman Coulter) | TRAP-6 60μM | >1300 sites |
| Philadelphia | not performed | not performed | not performed | not performed |
| Caceres | not performed | not performed | not performed | not performed |
| Coimbra | flow-cytometry (P-selectin) | FACS Calibur (Becton Dickinson) | AA 0.1 mM  TRAP 5 µM and 25 µM  ADP 5 µM  EPI 20 µM | Abnormal if % of positive cells <20% of control value |
| Detroit | not performed | not performed | not performed | not performed |
| Bergamo | not performed | not performed | not performed | not performed |

**δ-granule release**

|  | Method (kind of analyte) | Instrument name (company) | agonist (dose) | normal range |
| --- | --- | --- | --- | --- |
| Perugia | Lumiaggregometry (ATP) | Chronolog700 (Mascia Brunelli) | ADP 5 µM  EPI 100 µM  AA 1 mM  Coll 2 µg/ml | 0.39-0.86  0.36-0.92  0.45-1.30  0.57-1.2  nmoli/10^8^ plt |
| Marseille | not performed | not performed | not performed | not performed |
| Milan | Lumiaggregometry (ATP) | Chronolog 400VS (Mascia Brunelli) | ADP 2 µM  EPI 5 µM  TRAP 10 µM  Coll 2 µg/ml  U46619 1 µM  AA 1 mM | 0.049-0.908  0.069-1.026  0.191-1.267  0.343-1.157  0.153-0.914  0.223-1.127  nmoli/10^8^ plt |
| Buenos Aires | not performed | not performed | not performed | not performed |
| Rome | flow-cytometry (CD63) | FC500 (Beckman Coulter) | ADP 10μM PAR1-AP 25μM  ADP 10μM | Abnormal if MFI <50% of control value |
| Pessac | Lumiaggregometry (ATP)  Flow Cytometry (mepacrine) | Chronolog700  (Mascia Brunelli)  FC 500 (Beckman Coulter) | ADP 10 µM  Coll 2 µg/mL  TRAP 50 µM |  |
| Philadelphia | not performed | not performed | not performed | not performed |
| Caceres | not performed | not performed | not performed | not performed |
| Coimbra | Flow Cytometry (mepacrine) | FACS Calibur (Becton Dickinson) | TRAP6 30 uM | Abnormal if ratio MIF absortion/ MIF release < 1.5 |
| Detroit | not performed | not performed | not performed | not performed |
| Bergamo | not performed | not performed | not performed | not performed |

**Platelet GPIIb/IIIa expression (flow cytometry)**

|  | Instrument name (company) | Antibody anti GPIIb clone (company) | normal range (%) | normal range (MFI) | Antibody anti GPIIIa clone (company) | normal range (%) | normal range (MFI) |
| --- | --- | --- | --- | --- | --- | --- | --- |
| Perugia | FC500 (Beckman Coulter) | clone P2 (Beckman Coulter) | 90-100 | 22-30 | clone SZ21 (Beckman Coulter) | 90-100 | 16-24.9 |
| Marseille | FC500 (Beckman Coulter) | clone P2 (Beckman Coulter) | 90-100 | 23-40 | not performed | not performed | not performed |
| Milan | FACSLyric (Becton Dickinson) | Clone HIP2 and HIP8 (Becton Dickinson) | - | >10310 | VI-PL2 (Becton Dickinson) | - | >14754 |
| Buenos Aires | FACS Aria (Becton Dickinson) | Becton Dickinson | 96.6-99.9 | 0.77-1.23 | Becton Dickinson | 97.4-99.5 | 0.74-1.24 |
| Rome | FC500 (Beckman Coulter) | HIP8 (Becton Dickinson) | 63-90 | 3-6.4 | clone SZ21 (Beckman Coulter) | 90-100 | 16-24.9 |
| Pessac | FC500 (Beckman Coulter) | CD41 | 51000±14000 (number of sites) | not performed | not performed | not performed | not performed |
| Philadelphia | not performed | not performed | not performed | not performed | not performed | not performed | not performed |
| Caceres | not performed | not performed | not performed | not performed | not performed | not performed | not performed |
| Coimbra | FACS Calibur (Becton Dickinson) | kit Platelet Gp Screen (BioCytex) | Basal: 51.000±14.000 sites per cell  Activated: 85.000±27.000 sites per cell | Basal: 51.000±14.000 sites per cell  Activated: 85.000±27.000 sites per cell | kit Platelet Gp Screen (BioCytex) | Basal: 51.000±14.000 sites per cell  Activated: 85.000±27.000 sites per cell | Basal: 51.000±14.000 sites per cell  Activated: 85.000±27.000 sites per cell |
| Detroit | Gallios (Beckman Coulter) | clone P2 (Beckman Coulter) | >95 | 59 – 89 | clone SZ21 (Beckman Coulter) | >95 | 32 - 58 |
| Bergamo | FACSCanto (Becton Dickinson) | Serotec, clone PM6/248) | >90 | 300-600 | clone RUU-PL 7F12 (Becton Dickinson) | >90 | 80-180 |

**Platelet GPIIb/IIIa activation (flow cytometry)**

|  | Instrument name (company) | Antibody clone (company) | Agonist (dose) | normal range (%) | normal range (MFI) |
| --- | --- | --- | --- | --- | --- |
| Perugia | FC500 (Beckman Coulter) | PAC-1 (Becton Dickinson) | ADP 10μM | 57.8-78.3 | 4.5-8.2 |
| Marseille | FC500 (Beckman Coulter) | PAC-1 (Becton Dickinson) | TRAP 50μM  ADP 10μM | - | 3.9-9.0  5.4-16.2 |
| Milan | not performed | not performed | not performed | not performed | not performed |
| Buenos Aires | FACS Aria (Becton Dickinson) | PAC-1 (Becton Dickinson) | ADP 5uM  ADP 20uM  TRAP-6 20uM | 93.6-98 (ADP 20) | 0.77-1.23 (ADP 20) |
| Rome | FC500 (Beckman Coulter) | PAC-1 (Becton Dickinson) | PAR1-AP 25μM  ADP 10μM | 39-80 | 1.8-4.5 |
| Pessac | not performed | not performed | not performed | not performed | not performed |
| Philadelphia | not performed | not performed | not performed | not performed | not performed |
| Caceres | not performed | not performed | not performed | not performed | not performed |
| Coimbra | FACS Calibur (Becton Dickinson) | PAC-1 (Becton Dickinson) | TRAP 25μM  ADP 10μM | Abnormal if patient % of positive cells <20% of control value | Abnormal if patient % of positive cells <20% of control value |
| Detroit | not performed | not performed | not performed | not performed | not performed |
| Bergamo | not performed | not performed | not performed | not performed | not performed |

**Serum TXB2**

|  | Method | normal range |
| --- | --- | --- |
| Perugia | ELISA (Cayman Chemical) | 61-133 ng/10^8^plts |
| Marseille | not performed | not performed |
| Milan | ELISA (Cayman Chemical) | >60 ng/ml |
| Buenos Aires | not performed | not performed |
| Rome | not performed | not performed |
| Pessac | not performed | not performed |
| Philadelphia | not performed | not performed |
| Caceres | not performed | not performed |
| Coimbra | not performed | not performed |
| Detroit | not performed | not performed |
| Bergamo | not performed | not performed |

**Supplementary Table 3.** ISTH-BAT BS distribution in specific IPFDs

| **IPFD** | **N** | **Median ISTH-BAT BS**  **(IQR)** |
| --- | --- | --- |
| **Glanzmann thrombasthenia** | **16** | **13.5 (9-17.5)** |
| **Primary secretion defect** | **10** | **3.5 (4-6)** |
| **delta-storage pool deficiency** | **7** | **5 (4-6)** |
| **Biallelic Bernard Soulier syndrome** | **5** | **6 (0-19)** |
| **Familial platelet disorder associated with**  **myeloid malignancy** | **5** | **2 (0-7.5)** |
| **Gray platelet syndrome** | **5** | **10 (8.5-12.5)** |
| **Hermansky-Pudlak syndrome** | **4** | **3.5 (1.2-11)** |
| **Combined alpha-delta granule deficiency** | **4** | **8.5 (7.2-9)** |
| **Defect of the** **P2Y_12_ Purinergic Receptor** | **3** | **13 (2-18)** |
| **CalDAG-GEFI defect** | **3** | **22 (10-24)** |
| **Defect of the TP receptor** | **2** | **5 (4-6)** |
| **Defects of collagen receptors** | **2** | **2.5 (2-3)** |
| **cPLA_2_ deficiency** | **1** | **10** |
| **Platelet-type Von Willebrand Disease** | **1** | **11** |

**Supplementary Table 4.** Number and type of patients enrolled from each center

|  |  | Center  1 | Center  2 | Center 3 | Center  4 | Center  5 | Center  6 | Center  7 | Center  8 | Center  9 | Center  10 | Center  11 |
| --- | --- | --- | --- | --- | --- | --- | --- | --- | --- | --- | --- | --- |
|  | ***number of patients*** | 17 | 1 | 17 | 3 | 3 | 5 | 2 | 13 | 4 | 1 | 2 |
| Type of disorder | Glanzmann thrombasthenia | 4 | 1 | 4 |  | 1 | 1 |  | 2 | 1 |  | 2 |
|  | Primary secretion defect | 5 | 0 | 0 |  |  |  | 1 | 4 |  |  |  |
|  | delta-storage pool deficiency | 0 | 0 | 2 |  |  | 2 | 1 | 2 |  |  |  |
|  | Biallelic Bernard Soulier syndrome | 0 | 0 | 1 |  |  | 1 |  | 1 | 2 |  |  |
|  | Familial platelet disorder associated with  myeloid malignancy | 2 | 0 | 3 |  |  |  |  |  |  |  |  |
|  | Gray platelet syndrome | 0 | 0 | 1 |  | 2 |  |  | 1 |  | 1 |  |
|  | Hermansky-Pudlak syndrome | 2 | 0 | 0 | 2 |  |  |  |  |  |  |  |
|  | Combined alpha-delta granule deficiency | 1 | 0 | 3 |  |  |  |  |  |  |  |  |
|  | Defect of the P2Y_12_ Purinergic Receptor | 0 | 0 |  |  |  |  |  | 2 | 1 |  |  |
|  | CalDAG-GEFI defect | 0 | 0 | 3 |  |  |  |  |  |  |  |  |
|  | Defect of the TP receptor | 1 | 0 |  |  |  | 1 |  |  |  |  |  |
|  | Defects of collagen receptors | 1 | 0 |  | 1 |  |  |  |  |  |  |  |
|  | cPLA_2_ deficiency | 0 | 0 |  |  |  |  |  | 1 |  |  |  |
|  | Platelet-type Von Willebrand Disease | 1 | 0 |  |  |  |  |  |  |  |  |  |

**Supplementary Table 5.** Number of patients assessed for each laboratory test

| **Test** | **Number of assessed patients** |
| --- | --- |
| *Platelet count* | 68 |
| *MPV* | 65 |
| *LTA* |  |
| Adrenaline | 57 |
| ADP | 65 |
| Collagen | 67 |
| Arachidonic acid | 63 |
| TRAP6 | 40 |
| U46619 | 33 |
| Ristocetin | 39 |
| *PFA-100®* | 36 |
| *Bleeding time* | 30 |
| *α granule content/release* | 35/26 |
| *δ granule content/release* | 32/30 |
| *Clot retraction* | 31 |
| *Flow cytometry* |  |
| GPIIb | 37 |
| GPIIIa | 30 |
| GPIX | 26 |
| GPIbα | 51 |
| GPIa/IIa | 18 |
| PAC-1 | 31 |
| *Serum TxB_2_* | 17 |
| *TEM* | 8 |

**Supplementary Table 6.** Association of platelet function assays results and clinical symptoms as assessed by the X^2^ test

| **Test** | **Bleeding symptom** | **p** |
| --- | --- | --- |
| LTA in response to U46619 | Bleeding after surgery | 0.012 |
| LTA in response to ADP (high doses) | Menorrhagia | 0.014 |
| LTA in response to TRAP-6 (high doses) | Menorrhagia | 0.034 |
| PAC-1 binding upon activation with ADP | Bleeding after tooth extraction | 0.039 |
| Defective content of α−granules | Epistaxis | 0.026 |
| Defective content of α−granules | Bleeding after surgery | 0.045 |

**Supplementary figure 1**

**Supplementary Figure 1**. ISTH BAT BS of patient from each center. The percentage of pathological BS is reported in red

**Supplementary Figure 2**

**
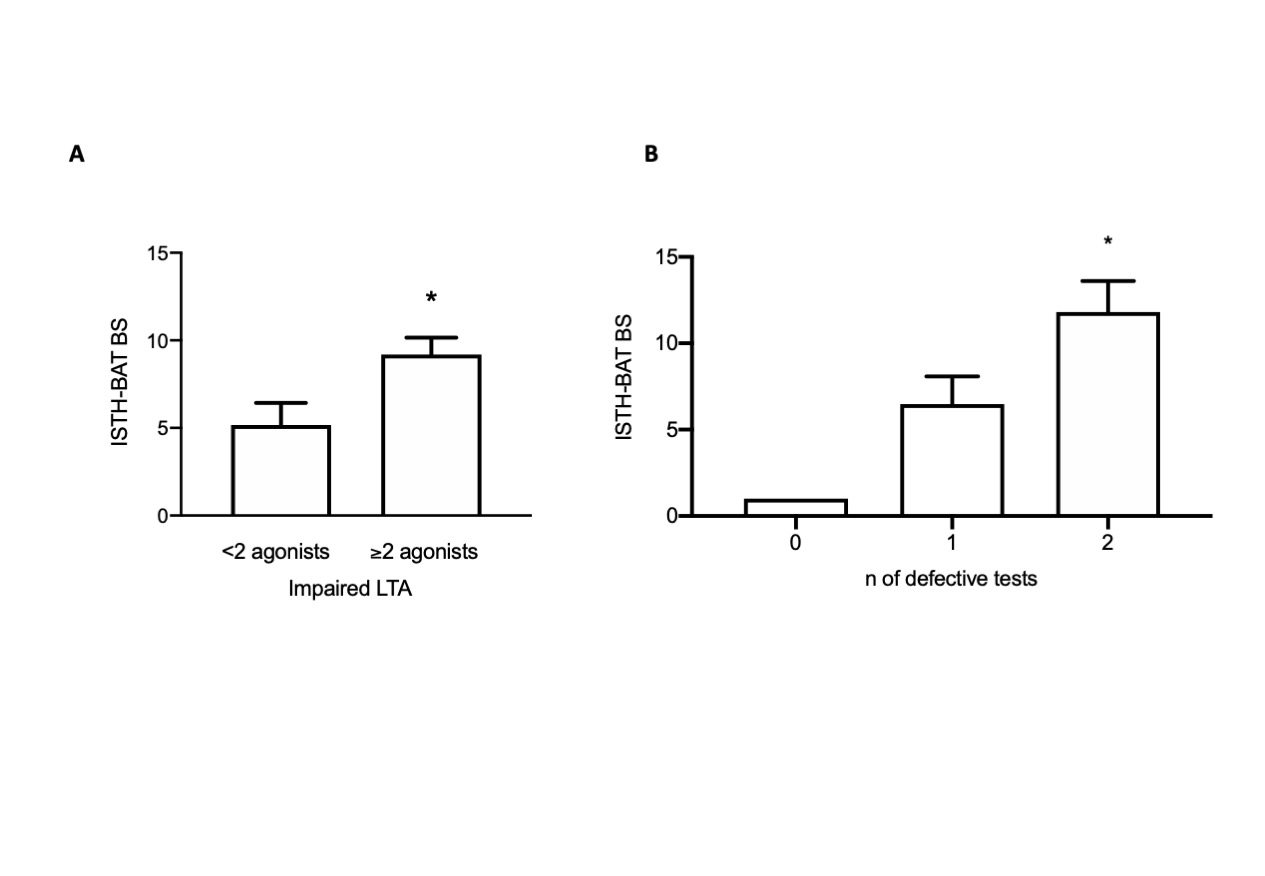
**

**Supplementary Figure 2.**

ISTH-BAT BS of patients with an impaired LTA in response to <2 or ≧ 2 agonists (*p<0.05, Mann-Whitney test)

**Supplementary Figure 3**


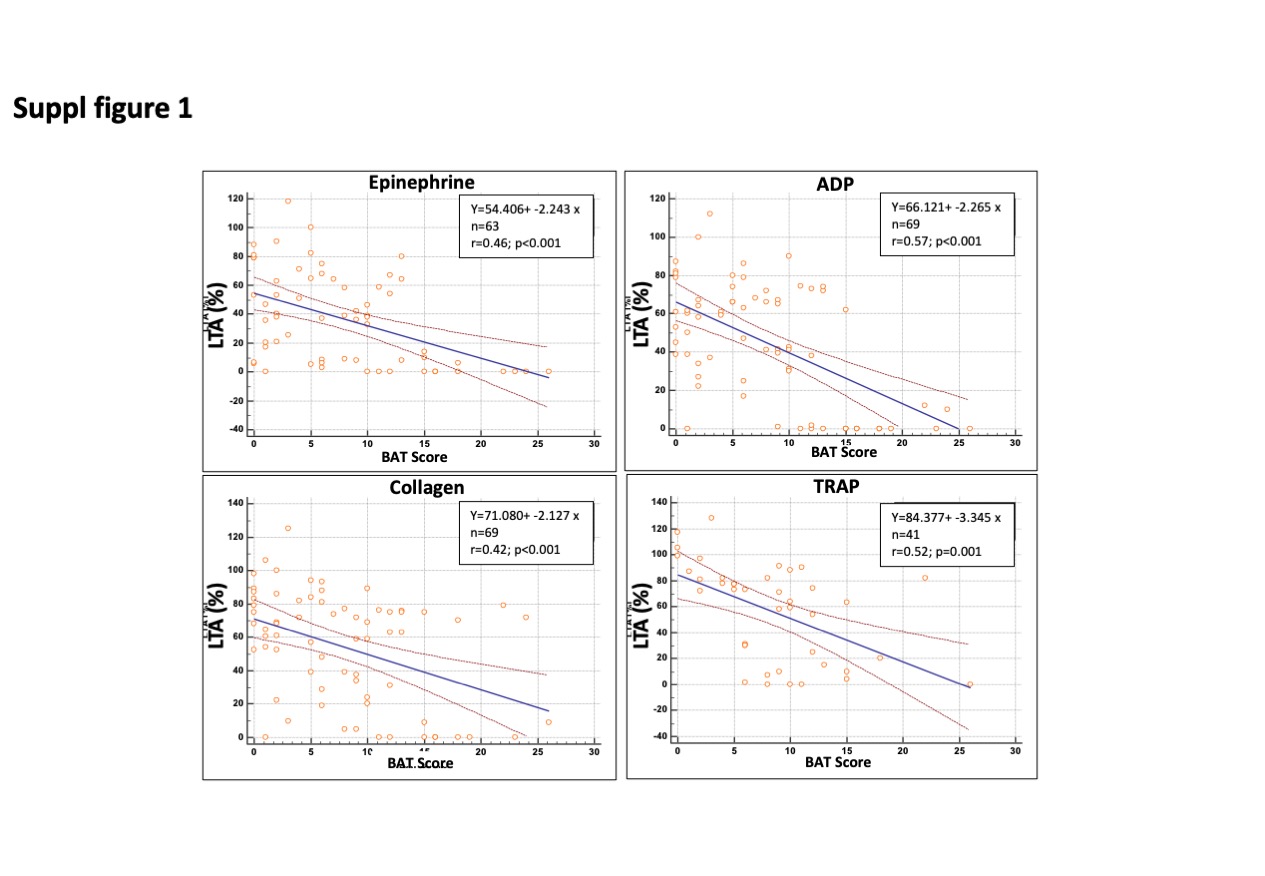


**Supplementary Figure 3**. Correlation between BS value and percentage of platelet aggregation induced by epinephrine, ADP, collagen and TRAP-6.

**Supplementary Figure 4**

**Supplementary Figure 4.** Percentage of platelet aggregation as assessed by LTA in patients with normal or high BS in response to low doses of different stimuli. Data are shown as violin plots, expressing median and quartiles and the frequency distribution curve and the individual points (*p<0.05 vs normal BS, **p<0.01 vs normal BS, One-way ANOVA). EPI=epinephrine.

**Supplementary Figure 5**


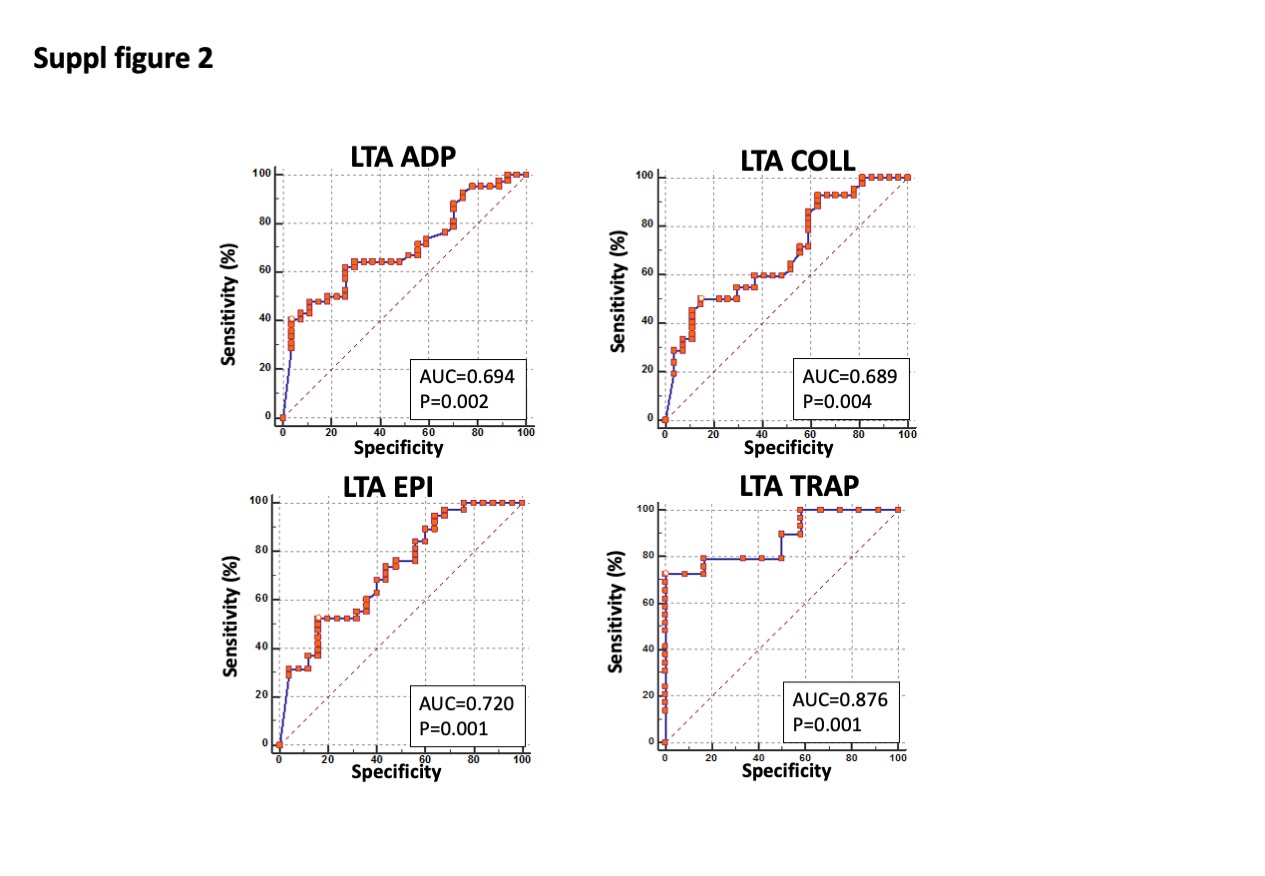


**Supplementary Figure 5**. ROC curves for the prediction of pathologic BS in subjects with a platelet aggregation defect in response to epinephrine (A), ADP (B), collagen (C) and TRAP-6 (D).

Cut off: ≤14 for EPI, ≤17 for ADP, ≤48 for Coll and ≤71 for TRAP.

Median normal range: EPI: 50.7-87, ADP:58.8-87.6, COLL: 68.9-91.9, TRAP: 72.5-90.9

**Supplementary Figure 6**


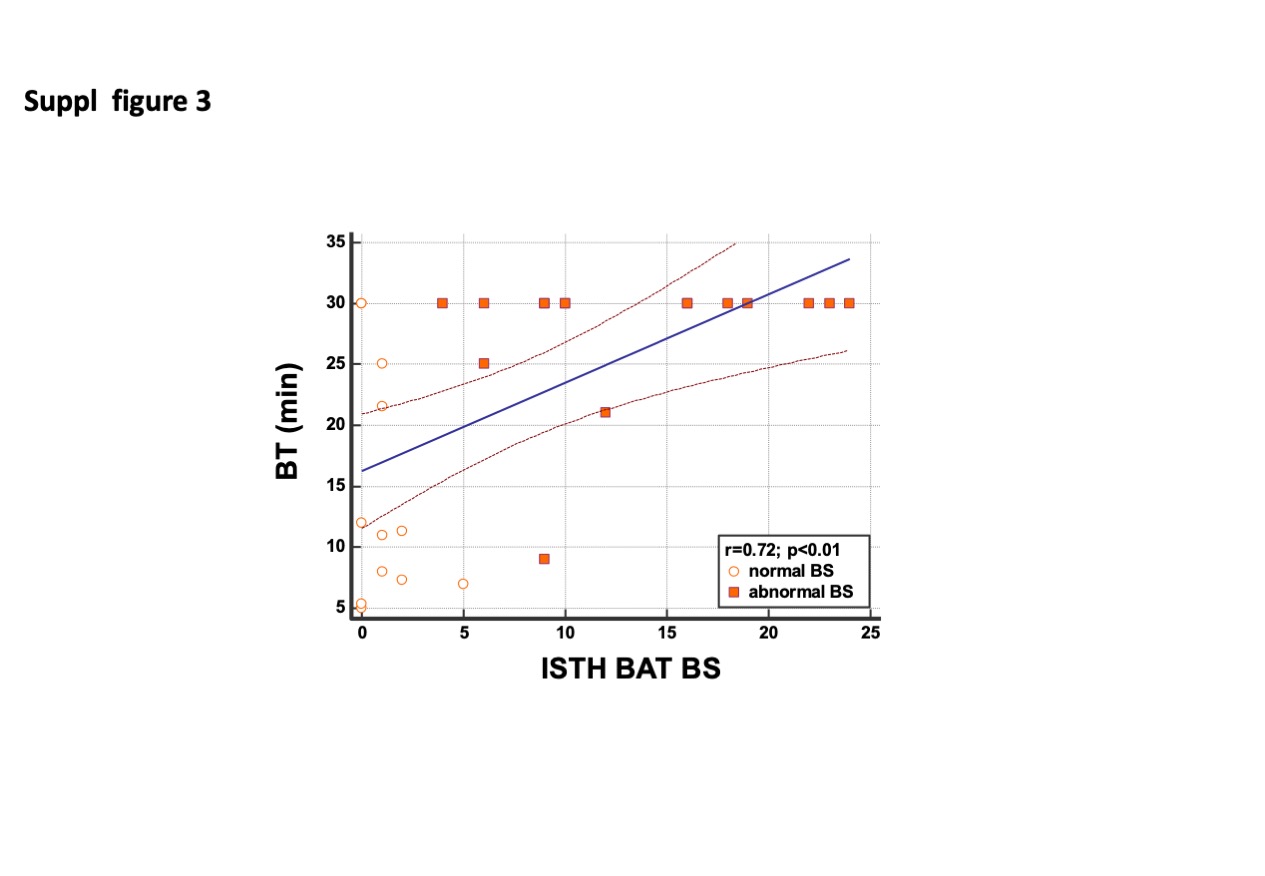


**Supplementary Figure 6**. Correlation between BT and BS value. r=0.72, p<0.01.

**Supplementary Figure 7**


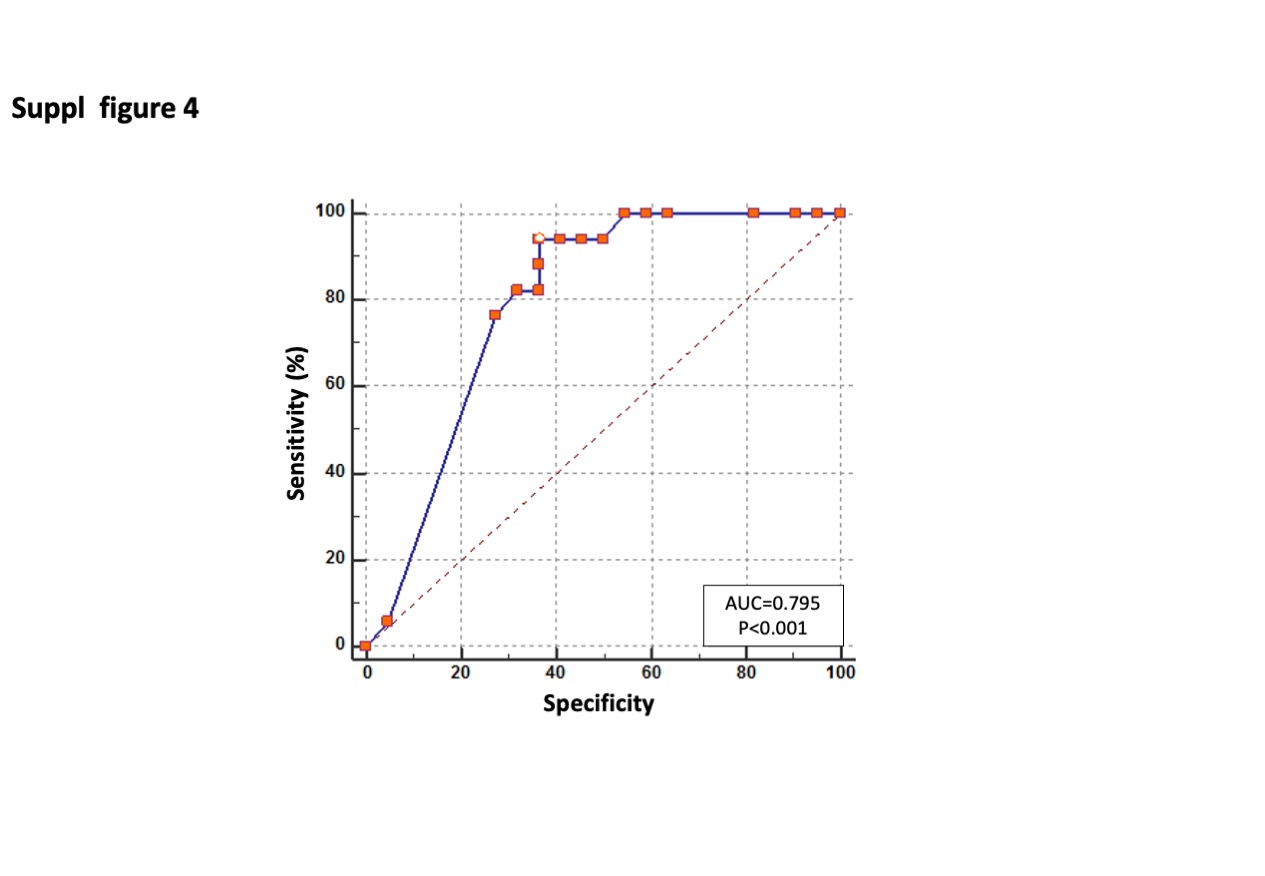


**Supplementary Figure 7**. ROC curve for the prediction of pathologic BS in subjects with a prolonged BT. Cut off >12 minutes. Median normal range: < 9 min.

**Supplementary Figure 8**


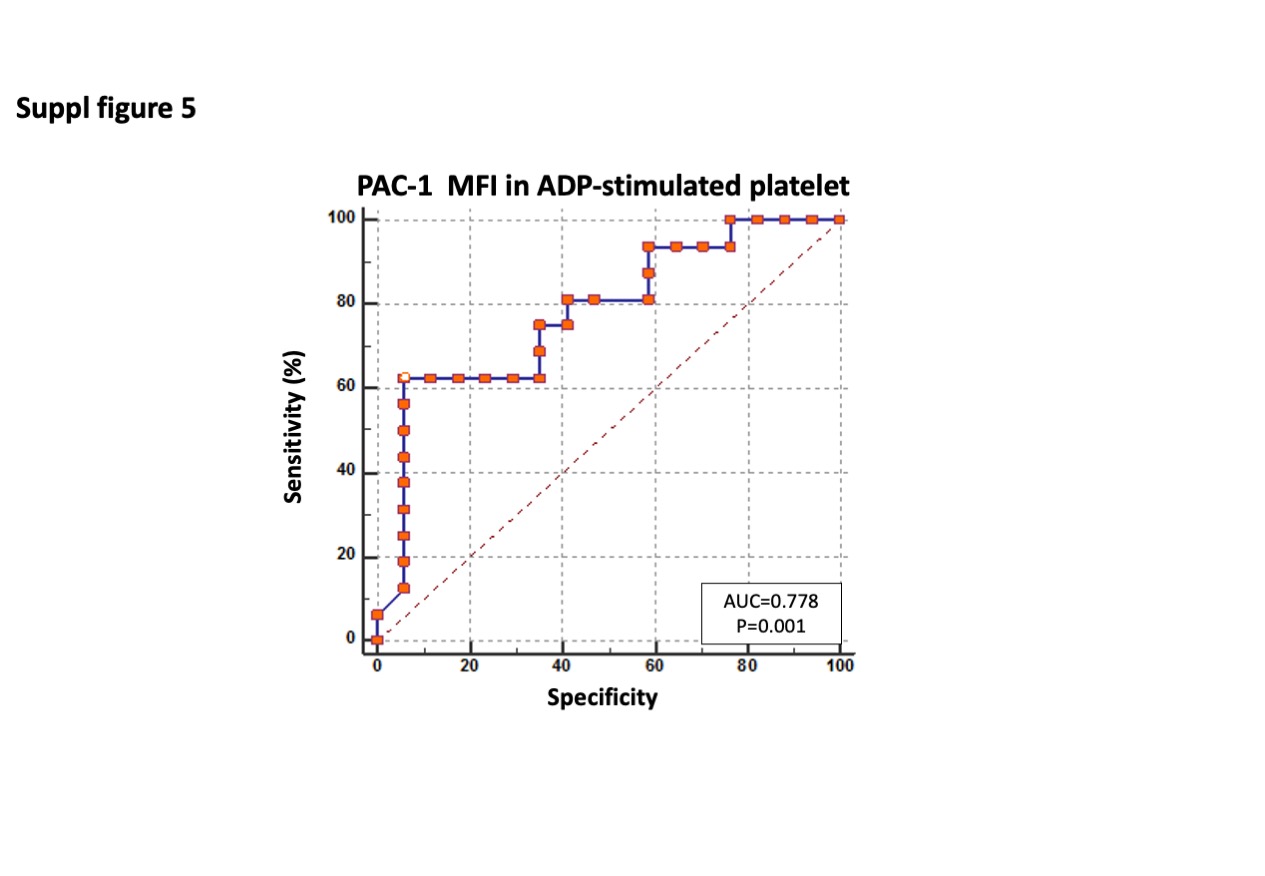


**Supplementary Figure 8**. ROC curve for the prediction of pathologic BS in subjects with a defective PAC-1 binding in response ADP. Cut off ≤3.7MFI. Median normal range: 5-13.2 MFI

**Supplementary Figure 9**

**Supplementary Figure 9.** ISTH-BAT BS of patients with normal LTA and α-granule content (0), defective LTA or α -granule content (1), defective LTA and α-granule content (2) (*p<0.05, Mann-Whitney test)
